# FACTORS ASSOCIATED WITH COMPLEX REGIONAL PAIN SYNDROME IN SURGICALLY TREATED DISTAL RADIUS FRACTURE

**DOI:** 10.1590/1413-785220172505165544

**Published:** 2017

**Authors:** JOEL ORTIZ-ROMERO, IGNACIO BERMUDEZ-SOTO, RUBÉN TORRES-GONZÁLEZ, FERNANDO ESPINOZA-CHOQUE, JESÚS ABRAHAM ZAZUETA-HERNANDEZ, JOSÉ MANUEL PEREZ-ATANASIO

**Affiliations:** 1. Department of Traumatology, Unidad Médica de Alta Especialidad “Dr. Victorio de la Fuentes Narváez”, Instituto Mexicano del Seguro Social, Mexico City, Mexico.; 2. Department of Education and Research, Unidad Médica de Alta Especialidad “Dr. Victorio de la Fuentes Narváez”, Instituto Mexicano del Seguro Social, Mexico City, Mexico.; 3. Department of Research, Unidad Médica de Alta Especialidad “Dr. Victorio de la Fuentes Narváez”, Instituto Mexicano del Seguro Social, Mexico City, Mexico.

**Keywords:** Radius fractures, Surgical procedures, operative, Reflex sympathetic dystrophy, Complex regional pain syndrome, Insurance beneficiary

## Abstract

**Objective:**

The aim of this study was to identify factors associated with developing complex regional pain syndrome (CRPS) after surgical treatment for distal radius fracture (DRF).

**Methods:**

This case-control study analyzed patients seen from January 2014 to January 2016. Results: In our sample of 249 patients, 4% developed CRPS. Associated factors were economic compensation via work disability (odds ratio [OR] 14.3), age (OR 9.38), associated fracture (OR 12.94), and level of impact (OR 6.46), as well as psychiatric history (OR 7.21).

**Conclusions:**

Economically-productive aged patients with a history of high-impact trauma and patients with a history of psychiatric disorders have greater risk of developing CRPS after DRF. ***Level of Evidence III, Case-Control Study.***

## RESUMO

**Objetivo:**

Este estudo tem como objetivo identificar fatores de risco associados ao desenvolvimento de síndrome de dor regional complexa (CRPS) após o tratamento cirúrgico da fratura distal do rádio (DRF).

**Métodos:**

Este estudo de caso/controle analisou pacientes atendidos de janeiro de 2014 a janeiro de 2016. Resultados: Em nossa amostra de 249 pacientes, 4% desenvolveram CRPS. Os fatores associados foram compensação econômica (razão de chances [RC] 14,3), idade (RC 9,38), fratura associada (RC 12,94) e nível de impacto (RC 6,46), bem como história psiquiátrica (RC 7,21).

**Conclusões:**

Os pacientes com idade produtiva e história de trauma de alto impacto e os com história de transtornos psiquiátrico têm maior risco de desenvolver CRPS depois de DRF. **Nível de Evidência III, Estudo de Caso Controle.**

## INTRODUCTION

The aim of this study was to identify risk factors associated with the development of complex regional pain syndrome (CRPS) in patients with distal radius fractures (DRF) treated surgically. DRF have been identified in humans since we have walked upright, but treatment from ancient times to the mid-twentieth century was similar and had a high rate of complications. Surgical management was introduced during the 1970s, and at the beginning of the 21st century various treatment options were offered.^1^ DRF is the most common human fracture (1/6 of all fractures) regardless of age.^2^ In the adult population, persons >60 years of age, postmenopausal women, and urban residents have a higher risk for DRF. Young adults are at lower risk for DRF, but this group is occupationally active and sequelae in this age group have more significant consequences.^2^ ^,^ ^3^

CRPS is a complication that can present as multiple injuries characterized by chronic, persistent pain (severe and debilitating) in the absence of cell damage. It is characterized by autonomic pain and sensory (allodynia, hyperesthesia), motor, trophic (osteopenia), and vasomotor changes (hyperemia and hyperthermia) that culminate in dysfunction of the extremity.^4^ Patients with DRF develop CRPS in 1-37% of cases, with a direct impact on quality of life, psychosocial wellbeing, and decrease in work capacity.^5^ ^,^ ^6^ CRPS varies in appearance and may occur immediately or weeks after trauma or surgical treatment;^4^ this syndrome is classified into type I (without evidence of nerve damage) and type II (with evidence of nerve injury).^7^ ^,^ ^8^

Risk factors for developing CRPS in the upper extremities have been studied extensively and associated with various surgical procedures such as dermofasciotomy and carpal tunnel release. DRF is associated in 30% of cases of CRPS in the upper extremities.^5^ ^,^ ^9^ Few studies mention risk factors for CRPS in patients who present DRF with surgical management.^6^ The objective of this study is to identify the demographic risk factors of the injury for developing CRPS in patients who received surgical treatment for DRF.

## MATERIALS AND METHODS 

A case-control study with non-probabilistic consecutive sampling was carried out in patients seen from January 2014 to January 2016 in order to identify factors associated with the development of CRPS after surgical treatment for DRF. This study was approved by the institutional review board (registration number R-2016-3401-24) and was carried out in accordance with the Declaration of Helsinki (1995). The results obtained are strictly confidential and were strictly for academic use. Informed consent was not required because the study collected secondary data (from medical records) and the natural history of the disease was not altered.

We included patients over 18 years of age with open or closed DRF who received surgical treatment and had a complete medical record and X-ray available in the digital system. We excluded patients who received surgical treatment in the emergency department because they did not have a complete medical record. The included patients developed pain after standard surgical treatment that was not alleviated by standard analgesia and were assessed by anesthesiologists specializing in pain management who diagnosed CRPS according to the Budapest criteria.^8,10^ The controls were patients who did not develop pain after surgical treatment, or who had a good response to standard analgesia.

A total of 249 patients were included in the study. All patients were treated surgically by experts in trauma and orthopedics with over 5 years of experience. After the surgery, follow-up was provided until consolidation. All cases were tracked via an electronic outpatient system until discharge or diagnosis with CRPS by the pain specialist. 

Risk factors related to CRPS are multifactorial and based on prior included studies,^6^ ^,^ ^11^ ^,^ ^12^ age >60 years, sex, exposed fracture, high-impact injuries (carpal luxation fracture, fracture of long bones, associated fracture of the humerus, femur, scapula, or craniofacial bones, loss of consciousness, motor vehicle injuries), low impact injuries (simple stroke, fall from own height), exposed fracture, high number of manipulations, presence of comorbidities, psychiatric history, type of fracture according to Fernandez classification, AO type of fracture, type of treatment, and number of manipulations. All surgeries were performed by orthopedic surgeons with >6 years surgical experience with DRF. Patients who were managed with closed reduction and external fixation (CREF) in addition to mixed osteosynthesis (MO) were kept immobilized until X-rays showed consolidation, after which the fixator was removed and the patient was referred to physical rehabilitation. In patients with open reduction and internal fixation (ORIF), antebrachial splint immobilization was maintained and subsequently removed systematically at 7-14 days to initiate active mobilization and rehabilitation exercises. Flexion and extension exercises of the elbow and fingers were indicated from the immediate postoperative period. After consolidation was corroborated via X-ray, all patients were sent to the physical and rehabilitation medicine department where they were given a physiotherapy form and instructions on how to perform the exercises at home. 

## RESULTS

A 4% incidence of CRPS (10 cases) was identified. Demographic characteristics of the population and results after calculating the odds ratio (OR) of the variables studied are summarized in Table 1. Table 2 summarizes the times related to each type of treatment. 

**Table 1:** Demographic characteristics and OR for dichotomous variables.

Variable	n	Cases CRPS (+) n:10	Controls CRPS (-) n: 239	OR	CI	P
Female	169	5	164	2.1867	0.614-7.781	0.227
Male	80	5	75			
Direct economic beneficiary	101	9	92	14.38	1.79-115.38	0.0121
Without economic	148	1	147			
<60 years of age	126	9	117	9.3846	1.170-75.233	0.035
>60 years of age	123	1	122			
Exposed fx	2	0	2	4.5238	0.204-100.287	0.339
Closed fx	247	0	247			
Associated fx	107	9	98	12.949	1.614-103.861	0.0159
Non associated fx	142	1	141			
High energy	51	6	45	6.466	1.751-23.872	0.005
Low energy	198	4	194			
Tx CREF	154	6	148	0.9223	0.253-3.356	0.9023
Tx ORIF	67	2	65	0.669	0.138-3.234	0.6173
Tx MO	28	2	26	2.048	0.412-10.165	0.3805
≥ 2 manipulations	59	3	56	1.4005	0.350-5.596	0.6337
One manipulation	190	7	183			
Diabetes mellitus	55	0	55	0.1583	0.009-2.744	0.2054
No Diabetes mellitus	194	10	184			
Arterial hypertension	82	0	82	0.0909	0.005-1.570	0.991
No arterial hypertension	167	10	157			
Psychiatric history	10	2	8	7.2188	1.315-39.606	0.0229
No psychiatric history	239	8	231			

OR, odds ratio; CI, confidence interval; Tx, treatment; fx, fracture; CREF, closed reduction external fixation; ORIF, open reduction internal fixation; MO, mixed osteosynthesis; RA, rheumatoid arthritis; CRI, chronic renal insufficiency.

**Table 2:** Surgical treatment

CREF (n=154)		
Variable	Mean	SD
Ischemia time (minutes)	3.34	15.85
Surgical time (minutes)	33.2	17.67
Immobilization time (days )	53.8	11.55
**ORIF (n=67)**		
**Variable**	**Mean**	**SD**
Ischemia time (minutes)	56.93	21.06
Surgical time (minutes)	62.6	24.33
Immobilization time (days )	32.3	23.79
**MO (n=28)**		
**Variable**	**Mean**	**SD**
Ischemia time (minutes)	71.54	32.89
Surgical time (minutes)	79.54	41.59
Immobilization time (days)	48.43	12.73

CREF, closed reduction and external fixation; ORIF, open reduction and internal fixation; MO, mixed osteosynthesis; SD, standard deviation.

## DISCUSSION

Reports on complications of DRF and its definitive surgical management describe a 3-25% incidence of CRPS.^13^ In this study we found an incidence of 4%, which is within the internationally reported values. 

We found that patients with surgically-managed DRF associated with development of CRPS were direct beneficiaries (received economic compensation for work disability), had fractures associated with another bone, were <60 years of age, had psychiatric history, and had injuries related to high-impact injuries. Multiple studies have reported that females have up to twice the risk of developing CRPS,^5^ ^,^ ^6^ ^,^ ^11^ ^,^ ^14^ but we found no difference between sexes in this study. Another study reported an association between advanced age and CRPS,^6^ with a mean age of 56 years, but in our study patients <60 years of age demonstrated a higher risk of developing CRPS.

Another study describing injuries associated with DRF focused on damage to the carpal bones, triangular fibrocartilaginous complex, and distal ulna; while this same study reported development of distal ulnar radial instability, carpal collapse, and residual pain, it did not describe CRPS.^15^ Additionally, 17.2% of a series of 721 patients presented an associated fracture with short-term follow-up but without reports of subacute or chronic complications such as CRPS.^16^ This differs from our results, where associated fractures were very common (frequency of 43%). Studies describing DRF and multiple fractures have not analyzed the relationship with CRPS.^6^ ^,^ ^7^ ^,^ ^11^ ^,^ ^17^ ^,^ ^18^ A study by Rozen et al.^19^ reviewing exposed DRFs did not mention higher risk for CRPS; we agree with this study because our patients with exposed fractures were not more likely to develop CRPS.

High-impact injuries have already been considered significant, with twice the risk for developing CRPS.^5^ ^,^ ^6^ We found similar results, although the probability of developing CRPS in our study was five times greater. As for type of treatment, some sources report that management with CREF increases the risk of developing CRPS vs. MO with volar plate.^20^ ^-^ ^22^ We did not find any difference among the three types of surgical treatment (CREF, ORIF, and MO with volar plate) in terms of developing CRPS. Longer immobilization time or prolonged times of bone consolidation have been reported to be associated with CRPS (immobilization typically lasts 6 to 8 weeks).^9^ Our patients who were immobilized >8 weeks did not demonstrate increased probability of developing CRPS.

History of psychiatric disease such as anxiety or depression has been associated with image simulation or with patients who are hyper-reactive to injuries and have a low pain threshold.^23^ Jellad et al.^11^ evaluated 90 patients with a history of anxiety or depression who received conservative management of DRF and did not find higher risk of CRPS. In our study, a positive association was found with a history of psychiatric disease, with a six-fold increase for development of CRPS.

This study analyzed a number of comorbidities such as chronic kidney disease, osteoporosis, history of cancer, asthma, pulmonary diseases, rheumatoid arthritis, hypothyroidism, Parkinson’s disease and Herpes Zoster infection. Nevertheless, none of these comorbidities indicated a significant relation to developing CRPS.

One limitation of this study is the follow-up time for the patients managed surgically. After radiographic confirmation of consolidation, the patients were discharged and referred to a rehabilitation center along with follow-up by the family physician, and CRPS may consequently be clinically underdiagnosed. Another limitation of the study is the sample size and retrospective design. 

## CONCLUSION

Patients who may have a secondary benefit from prolonged disability due to DRF have a higher risk of developing CRPS. In addition, patients <60 years of age with associated fractures are closely related with high-impact fractures, generally from automobile accidents, falls from height, and sports, and have an elevated risk of developing CRPS. Patients with a psychiatric history have a greater risk of developing CRPS. 
